# Impaired Working Memory Updating for Emotional Stimuli in Depressed Patients

**DOI:** 10.3389/fnbeh.2018.00065

**Published:** 2018-04-04

**Authors:** Dandan Zhang, Hui Xie, Zhenhong He, Zhaoguo Wei, Ruolei Gu

**Affiliations:** ^1^CAS Key Laboratory of Behavioral Science, Institute of Psychology, Beijing, China; ^2^Shenzhen Key Laboratory of Affective and Social Cognitive Science, Shenzhen University, Shenzhen, China; ^3^Department of Clinical Psychology, Shenzhen Kangning Hospital, Shenzhen, China; ^4^Department of Psychology, University of Chinese Academy of Sciences, Beijing, China

**Keywords:** depression, working memory, emotion, updating, N-back task

## Abstract

Although two previous studies have demonstrated that depressed individuals showed deficits in working memory (WM) updating of both negative and positive contents, the effects were confounded by shifting dysfunctions and the detailed neural mechanism associated with the failure in N-back task is not clear. Using a 2-back task, the current study examined the WM updating of positive, negative and neutral contents in depressed patients. It is found that depressed patients performed poorer than healthy controls only when updating positive material. Using event-related potential (ERP) technique, the current study also investigated the neural correlates of updating deficits in depression. According to previous studies, the n-back task was divided into three sub-processes, i.e., encoding, matching and maintaining. Our ERP results showed that depressed patients had smaller occipital P1 for positive material compared to healthy controls, indicating their insensitivity to positive items on early encoding stage. Besides, depressed patients had larger frontal P2 and parietal late positive potential (LPP) than healthy controls irrespective of the valence of the words, reflecting that patients are inefficient during matching (P2) and maintaining (LPP) processes. These two mechanisms (insufficient attention to positive stimuli and low efficiency in matching and maintaining) together lead to the deficits of WM updating in depression.

## Introduction

It is well established that major depressive disorder (MDD) is associated with altered cognitive control which may contribute to symptoms such as anhedonia and maladaptive rumination (Marazziti et al., 2010; Rock et al., 2014). In particular, it has been proposed that impaired function of working memory (WM) is a hallmark of cognitive control deficits in depression (Austin et al., 2001; Joormann et al., 2011; Baddeley, 2013). Since WM has limited capacity, task-irrelevant information should be excluded from the systemso to ensure an efficient performance of the system (Baddeley and Hitch, 1974; Baddeley, 2003). It has been demonstrated that depressed individuals can hardly inhibit the interference of negative information. In particular, they not only fail to prevent irrelevant emotional information from entering WM, but also have difficulties in removing task-irrelevant negative information from WM (Joormann, 2004, 2010; Goeleven et al., 2006; Foland-Ross et al., 2013). As a result, excessive negative information is stored in their brain, contributing to uncontrollable and unintentional recurrence of negative thoughts and memories (Gotlib and Joormann, 2010; Joormann and Quinn, 2014).

It has been well known that the central executive system of WM is associated with three cognitive components, namely inhibition, shifting and updating (Baddeley and Hitch, 1974; Miyake et al., 2000). Specifically, inhibition refers to one’s ability to stop dominant or prepotent responses deliberately when necessary, or to suppress the interference of task-irrelevant information. Shifting concerns the ability to switch between tasks or reallocate attention between different mental sets. Updating function involves monitoring and dynamical manipulation of WM contents (Miyake et al., 2000). Using tasks such as Stroop and Go-NoGo, previous studies have demonstrated the neural mechanisms underlying the impaired inhibition function in depression (Kaiser et al., 2003; Wagner et al., 2006; Boggio et al., 2007; Mitterschiffthaler et al., 2008). Besides, numerous prior studies using Wisconsin Card Sorting Test and task-switching paradigm revealed a shifting impairment in depression (Channon, 1996; Merriam et al., 1999; Harvey et al., 2004; Rogers et al., 2004; Meiran et al., 2011). While inhibition and shifting deficits are often reported, the updating deficit in depression has been less concerned. Considering that updating is the major cognitive component of WM (Miyake et al., 2000; Harvey et al., 2004), and that among the three cognitive components, only updating deficit has been found correlated with the number of hospitalizations and longitudinal measures of depression severity (Harvey et al., 2004), it is important to clarify the updating deficits in depression so to comprehensively understand the impaired WM in the pathology of depression.

The N-back is a paradigm being widely used to investigate the updating process of WM (Owen et al., 2005; Redick and Lindsey, 2013). In this paradigm, a series of items are sequentially presented, and participants are required to answer whether the current item matches the one that presented N items earlier in the sequence. Using this paradigm, behavioral studies demonstrated that WM updating of non-emotional material is impaired in MDD patients (Nebes et al., 2000; Harvey et al., 2004), even when they are remitted (Nebes et al., 2000). So far as we know, there are only two studies that used the n-back task to investigate the WM updating of emotional contents in depressed participants (Levens and Gotlib, 2010, 2015). Using emotional stimuli, Levens and Gotlib (2010) found that current depression was associated with positive attenuation (rapid disengagement and difficult maintenance of positive stimuli) and negative enhancement (quick integration and slow disengagement of negative stimuli) in WM system. Moreover, the observed emotional bias in WM updating persisted beyond the depressive episode, which is believed to be related to the recurrent nature of depression (Levens and Gotlib, 2010). However, in these two studies, the faces with different emotional valences were presented in a random order across trials, thus the reported effects were associated with impaired WM shifting *per se*. Therefore, the first goal of the current study is to examine WM updating of negative and positive stimuli in depression using the n-back task with a block design. Furthermore, previous WM studies revealed inconsistent results regarding emotional contents: while Levens and Gotlib (2010, 2015) demonstrated that depressed individuals have difficulties in updating both positive and negative material in WM, some studies showed that depressed participants are difficult to update only negative items in WM tasks (Joormann et al., 2011). Thus the second goal of the current study is to examine whether updating deficit in WM is specific to negative content in depression.

This study employed the event-related potential (ERP) technique to elucidate specific temporal dynamics that involved in the updating function of WM in appreciation of its exquisite temporal resolution. According to previous studies, the N-back task mainly involves three cognitive processes, namely encoding, matching and maintaining (Chen et al., 2008; Barbey et al., 2013). Specifically, participants need to encode the currently presented item and match it with the item that presented N items earlier; meanwhile, target items should be maintained in WM system for N trials. Previous memory studies have suggested three ERP components that might reflect the three cognitive processes in N-back. First, the occipital P1 is associated with attentional involvement and fast encoding of visual stimuli (Clark and Hillyard, 1996; Hillyard and Anllo-Vento, 1998; Pérez-Edgar et al., 2006). Thus the amplitude of P1 is the index of encoding process in N-back task. Second, the frontal P2 reflects top-down matching procedure that compares the encoded sensory inputs and memory representations (Luck and Hillyard, 1994; Federmeier et al., 2005; Evans and Federmeier, 2007; Freunberger et al., 2007). Third, the parietal late positive potential (LPP) has been widely documented to be linked to sustained processing and maintenance (retention) of information in memory system (Ruchkin et al., 1990; Cuthbert et al., 2000; Weinberg and Hajcak, 2011; Hajcak et al., 2012; Auerbach et al., 2015; Lewis et al., 2015). Based on these knowledge, the current study plans to compare the three ERP components between depressed patients and healthy controls. It is hoped to reveal the impaired functions in patients associated with the three cognitive procedures (encoding, matching and maintaining) in n-back task. Since there has been no previous study providing relevant information on this topic, no specific hypothesis was made regarding the ERP components.

## Materials and Methods

### Participants

The depressed group contained 26 outpatients with MDD recruited from clinics in Shenzhen Kangning Hospital. Meantime, a total of 25 healthy adults were recruited as control group through advertisements in the community. There was no significant difference between the two groups with regard to age, handedness and education (Table 1).

**Table 1:** Demographic and clinical data of depressed and control groups.

Characteristics	Depressed patients (*n* = 26)	Control subjects (*n* = 25)	Statistics
Mean age, years	37.4 (21–57)	37.6 (23–56)	*t*_(49)_ = −0.09, *p* = 0.930
Education time, years	14.0 (6–19)	13.3 (9–22)	*t*_(49)_ = 0.81, *p* = 0.419
Sex, male/female	13/13	12/13	
Handedness, right/left	26/0	25/0	
BDI-II	21.0 (14–48)	2.1 (0–5)	*t*_(49)_ = 12.21, *p* < 0.001
STAI-T	42.7 (23–65)	41.0 (20–53)	*t*_(49)_ = −0.48, *p* = 0.617
Duration of illness, months	23.9 (0.5–240.0)		
Age at disease onset, years	34.8 (21–41)		
Number of lifetime episodes	2.1 (1–5)		

*Descriptive data are presented as mean (range). BDI-II, Beck Depression Inventory Second Edition. STAI-T, the Trait form of Spielberger’s State-Trait Anxiety Inventory*.

Patients were diagnosed with a current major depressive episode according to the Diagnostic and Statistical Manual (DSM-IV; American Psychiatric Association, 1994). The diagnosis was based on structured clinical interview for DSM (SCID; First et al., 1996a) and chart review. In addition, all MDD participants were with a score of ≥14 on the Beck Depression Inventory Second Edition (BDI-II; Beck et al., 1996) in the time of experiment. Exclusion criteria were neurological disorders and any comorbid Axis I and Axis II disorders. The interview and clinical symptom rating were based on consensus of two senior psychiatrists (Zhaoguo Wei and Xinying Li) who were trained with a relatively high reliability (*κ* = 0.86). At the time of experiment, the 26 patients were either untreated with any antidepressant medication, or had undergone a wash-out period of at least 4 weeks.

Healthy control participants were screened for current Axis I and II disorders using the SCID-I/NP (First et al., 2002) and SCID-II (First et al., 1996b), and they were additionally required to have a BDI-II score of ≤5.

Exclusion criteria for both MDD and control participants were: (1) seizure disorder; (2) history of head injury with possible neurological sequelae; and (3) substance abuse or dependence in the past 6 months.

This study was carried out in accordance with the recommendations of the ethical guidelines of the American Psychological Association (2002) with written informed consent from all subjects. All subjects gave written informed consent in accordance with the Declaration of Helsinki. The protocol was approved by the Ethics Committee of Shenzhen Kangning Hospital.

### Experimental Design and Stimuli

A three (valence of material: negative/neutral/positive) by two (group: depressed patients vs. nondepressed controls) design was used in this study.

A total of 60 adjectives, each consisted of two Chinese characters, were selected from the Chinese Affective Words System (Wang et al., 2008) as the stimuli, with each emotion category (negative, neutral and positive) containing 20 items. Examples of negative adjectives are selfish, cowardly and dangerous. Examples of positive adjectives are smart, beautiful and elegant. Examples of neutral adjectives are busy, smooth and compact. The material had been assessed for its valence and arousal on a 9-point scale by a large sample of Chinese participants in a previous survey. The three categories of words differed significantly in valence (negative = 2.79 ± 0.16, neutral = 4.86 ± 0.66; positive = 7.10 ± 0.14, *F*_(2,57)_ = 585.6, *p* < 0.001, pairwise comparisons: *p*s < 0.001). The arousal of neutral words (4.31 ± 0.56) were significantly lower than negative (5.33 ± 0.62) and positive (5.15 ± 0.50) words (*F*_(2,57)_ = 18.4, *p* < 0.001), while there was no significant difference between the arousal of negative and positive words (*p* = 0.966).

### Procedure

Since the current study is a pilot study, we did not consider the memory load in n-back task (see also Kessel et al., 2016). Besides, our behavioral pretest indicated that a memory load of two (*n* = 2) is the most suitable task for depressed patients, because 1-back led to ceiling effect and 3-back led to floor effect in this population. Therefore, the current study used a 2-back task to investigate the WM updating of emotional words in depression (see also Levens and Gotlib, 2010, 2015). The emotional 2-back task was composed of three blocks (negative, neutral and positive blocks). The order of the blocks was counterbalanced across subjects. Each block contained 60 trials, with half of the trials containing “match” items and the other half containing “mismatch” items.

**Figure 1:**
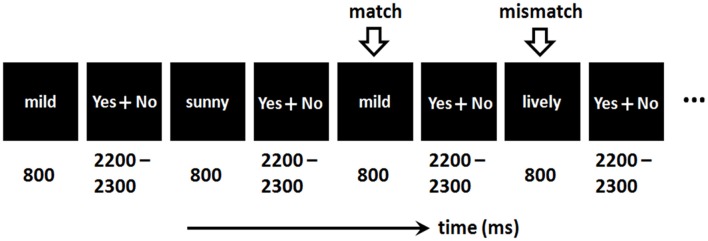
Illustration of the emotional 2-back task.

As shown in Figure 1, a word was presented for 800 ms in each trial. Then participants were required to respond as quickly as possible regarding whether the current word matched the one presented two trials earlier, by pressing the “F” or “J” button on the computer keyboard with their left or right index finger. The assignment of keys to “yes” and “no” answers was counterbalanced across participants.

### Electroencephalography (EEG) Recording and Analysis

Brain electrical activity was recorded by a 32-channel amplifier with a sampling frequency of 250 Hz (Brain Products, Gilching, Germany). Data were on-line recorded referentially against left mastoid and off-line re-referenced to “infinity” where the potential is zero (Yao, 2001; Tian and Yao, 2013; Dong et al., 2017). Electroencephalography (EEG) data were collected with electrode impedances kept below 5 kΩ. Ocular artifacts were removed from EEGs using a regression procedure implemented in NeuroScan software (Scan 4.3).

The recorded EEG data were filtered (0.01–30 Hz) and segmented beginning 200 ms prior to the onset of stimulus and lasting for 1000 ms. All epochs were baseline-corrected with respect to the mean voltage over the 200 ms preceding the onset of words, followed by averaging in association with experimental conditions. Epochs containing artifacts exceeding ±150 μV were rejected. The trial number in each condition is listed in Table 2. No significant difference of trial numbers was found between groups. One EEG dataset in depressed group was invalid due to technique problems. Therefore the final sample size for ERP analysis was 50 participants (25 patients and 25 healthy controls).

**Table 2:** Number of event-related potential (ERP) trials in each condition.

Condition	Depressed patients (*n* = 25)	Control subjects (*n* = 25)	Statistics
Negative words	34.8 ± 3.3	34.2 ± 3.5	*t*_(48)_ = 0.71, *p* = 0.842
Neutral words	33.3 ± 5.4	36.2 ± 5.1	*t*_(48)_ = −0.95, *p* = 0.347
Positive words	33.5 ± 4.2	36.5 ± 3.6	*t*_(48)_ = −1.21, *p* = 0.213

*Trials with large artifact or a incorrect response were excluded. Descriptive data are presented as mean ± standard deviation*.

This study focused on the three components (P1, P2 and LPP) elicited by negative, neutral and positive words in the two groups. Time windows for mean amplitude calculation were centered at the peak latencies of ERP components in grand-mean waveforms, with a shorter window length for early components and a longer length for late components. In ERP literature, the P1 component is often analyzed with the hemisphere (left/right) as a within-subject factor. However, since no hypothesis is made concerning the hemisphere distribution in this study, the mean P1 amplitude (time window = 100–140 ms) was calculated using the average amplitude at the symmetrical electrode sites (O1 and O2) to obtain a high signal-to-noise ratio (Luck and Gaspelin, 2017; see also Eldar et al., 2010; Raz et al., 2014; Liu et al., 2015; Zhang et al., 2016; Hammerschmidt et al., 2017). The P2 amplitude was calculated as the average amplitude at the electrode sites of Cz, FC1, FC2, FCz and Fz between 160–220 ms. The LPP amplitude was calculated as the average amplitude at the electrode sites of Pz, Cz, CP1 and CP2 between 350–600 ms. Amplitudes of ERP components at electrodes of interest are reported in Supplementary Material.

### Statistics

Descriptive data were presented as mean ± standard deviation, unless otherwise mentioned. The significance level was set at 0.05.

Repeated-measures ANOVA was performed on behavioral and ERP measurements, with the emotion category of words (negative/neutral/positive) as the within-subject factor, and group (depressed vs. control) as the between-subjects factor. Significant interactions were analyzed using simple effects model. Greenhouse-Geisser correction for ANOVA tests was used whenever appropriate. *Post hoc* testing of significant main effect and multiple comparisons were conducted using Bonferroni method.

## Results

### Behavioral Data

#### Accuracy

The main effect of the emotion category of words was significant (*F*_(2,98)_ = 11.9, *p* < 0.001, $\eta_{\text{p}}^{2}$ = 0.195). The accuracy in the negative condition (67.7 ± 18.4%) was significantly lower than that in the positive (74.8 ± 17.9%, *p* < 0.001) and neutral conditions (73.7 ± 18.8%, *p* = 0.005).

The interaction of emotion category by group was significant (*F*_(2,98)_ = 3.47, *p* = 0.035, $\eta_{\text{p}}^{2}$ = 0.066). In the positive condition, the control group (80.8 ± 13.9%) had significantly higher accuracy than the depressed group (69.1 ± 19.6%; *F*_(1,49)_ = 6.00, *p* = 0.018). However, this group differences did not achieve significant level in the neutral (control = 77.1 ± 17.6%, depressed = 70.4 ± 19.7%; *F*_(1,49)_ = 1.65, *p* = 0.205) and negative conditions (control = 69.4 ± 18.9%, depressed = 66.0 ± 18.2%; *F*_(1,49)_ < 1).

#### Reaction Time

The interaction of emotion category by group was significant (*F*_(2,98)_ = 4.86, *p* = 0.014, $\eta_{\text{p}}^{2}$ = 0.090). In the positive condition, the reaction time of patients (555 ± 129 ms) was significantly longer than that in control subjects (476 ± 143 ms; *F*_(1,49)_ = 3.34, *p* = 0.042). However, this group difference did not achieve significant level in neutral (*F*_(1,49)_ = 1.48, *p* = 0.229; control = 494 ± 164 ms, depressed = 561 ± 224 ms) and negative conditions (*F*_(1,49)_ = 1.48, *p* = 0.230; control = 592 ± 144 ms, depressed = 533 ± 194 ms).

### ERPs

#### Occipital P1

**Figure 2:**
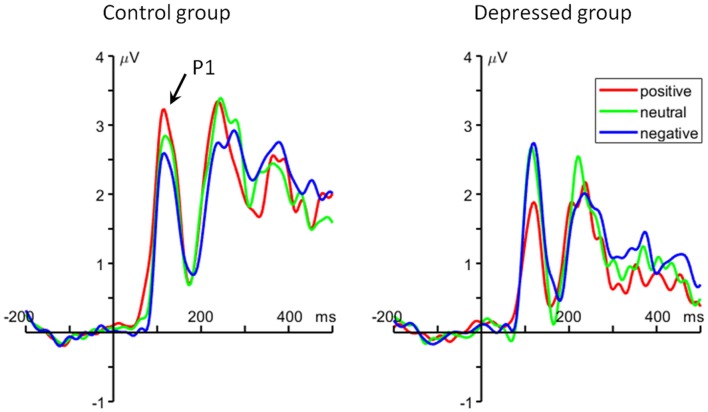
The grand average event-related potential (ERP) waveforms averaged at O1 and O2 electrodes, showing the occipital P1 component.

For the average amplitude, the interaction of emotion category by group was significant (*F*_(2,96)_ = 8.48, *p* = 0.001, $\eta_{\text{p}}^{2}$ = 0.150). For the positive words, the healthy control group (2.86 ± 1.69 μV) elicited larger P1 amplitude than MDD patients (1.78 ± 1.69 μV; *F*_(1,48)_ = 5.14, *p* = 0.028). However, there is no significant difference between groups on the P1 amplitude for the neutral (depressed group = 2.64 ± 1.70 μV, control group = 2.56 ± 1.85 μV; *F*_(1,48)_ < 1) and negative (depressed group = 2.68 ± 1.85 μV, control group = 2.32 ± 2.13 μV; *F*_(1,48)_ < 1) words (Figure 2).

For the peak latency, neither the main effect of emotion category (*F*_(2,96)_ = 3.05, *p* = 0.053, $\eta_{\text{p}}^{2}$ = 0.062) nor the main effect of group (*F*_(1,48)_ = 2.40, *p* = 0.128, $\eta_{\text{p}}^{2}$ = 0.048) was significant. The interaction of emotion category by group was not significant (*F*_(2,96)_ = 1.48, *p* = 0.234, $\eta_{\text{p}}^{2}$ = 0.031).

#### Frontal P2

**Figure 3:**
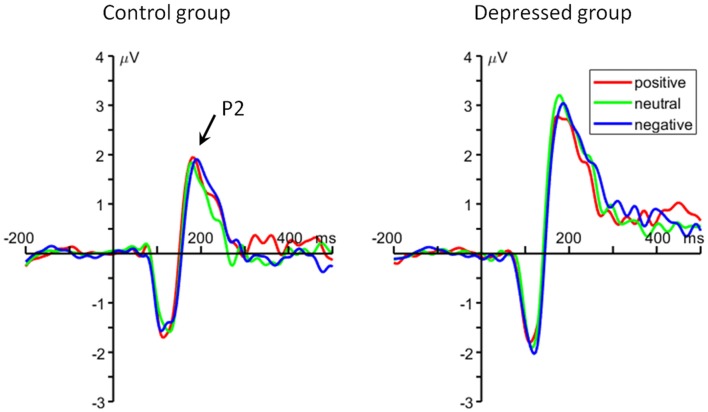
The grand average ERP waveforms averaged at Cz, FC1, FC2, FCz and Fz electrodes, showing the frontal P2 component.

For the average amplitude, the main effect of group was significant (*F*_(1,48)_ = 7.22, *p* = 0.013, $\eta_{\text{p}}^{2}$ = 0.131). MDD patients had larger P2 amplitudes (2.66 ± 1.96 μV) than control group (1.43 ± 1.57 μV; Figure 3).

For the peak latency, neither the main effect of emotion category (*F*_(2,96)_ < 1, $\eta_{\text{p}}^{2}$ = 0.003) nor the main effect of group (*F*_(1,48)_ < 1, $\eta_{\text{p}}^{2}$ = 0.013) was significant. The interaction of emotion category by group was not significant (*F*_(2,96)_ < 1, $\eta_{\text{p}}^{2}$ = 0.003).

#### Parietal LPP

**Figure 4:**
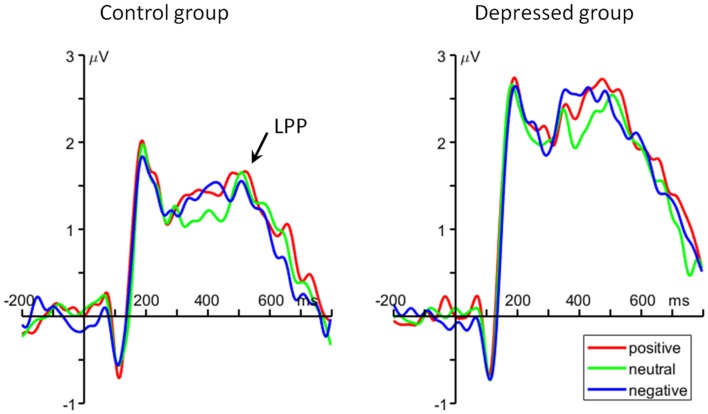
The grand average ERP waveforms averaged at Pz, Cz, CP1 and CP2 electrodes, showing the parietal late positive potential (LPP) component.

For the average amplitude, the main effect of group was significant (*F*_(1,48)_ = 6.37, *p* = 0.015, $\eta_{\text{p}}^{2}$ = 0.117). MDD patients had larger LPP amplitudes (2.32 ± 1.79 μV) than control group (1.37 ± 1.42 μV; Figure 4).

## Discussion

Using an emotional 2-back task, the present study explored the deficits of WM updating in MDD patients. Behavioral results showed a positive-specific deficit in the MDD group which is in line with the cognitive model of depression indicating that depression is associated with reduced response to positive stimuli (Disner et al., 2011). Our ERP results further demonstrated that depressed patients had reduced P1 amplitudes in response to positive words compared with control subjects, indicating that MDD patients are insensitive to positive stimuli in the early encoding stage of WM updating.

The current result mainly demonstrated a positive-specific impairment in depression. Our finding supported the notion in Beck’s cognitive model of depression (Disner et al., 2011) that depressed people show low sensitivity to positive stimuli, i.e., they pay little attention to positive material and have difficulty in remembering positive events. For instance, previous studies have shown that depressed participants spend less time to view happy facial expressions, relative to nondepressed individuals (Duque and Vázquez, 2015); depressed individuals exhibit reduced brain activities when processing positive relative to negative and neutral stimuli (Shestyuk et al., 2005; Epstein et al., 2006); they are unable to sustain engagement of neural circuits involved in positive affect and reward during an emotion regulation task (Heller et al., 2009). In contrast to depressed individuals, healthy adults usually show an enhanced processing for positive information (McCabe and Gotlib, 1995; Shane and Peterson, 2007; Sanchez and Vazquez, 2014), as indicated by behavioral and ERP (P1) results of this study. The most important finding here is the reduced P1 amplitudes for positive words in patients, which is consistent with our behavioral results, i.e., MDD patients had lower accurate rate and longer response time for positive words compared to control subjects. Given that the occipital P1 component is known to be correlated with early bottom-up selective attention (Luck et al., 1990; Clark and Hillyard, 1996; Hillyard and Anllo-Vento, 1998; Pérez-Edgar et al., 2006), our result indicates that positive words attracted less early attention during memory encoding in depressed subjects compared to control subjects. While a very similar P1 pattern has also been reported by previous studies using other tasks (Dai and Feng, 2011; Zhang et al., 2016), it should be pointed out that this is the first report of insufficient encoding of positive material during WM updating in depression.

At the meantime, the ERP data showed larger P2 and LPP amplitudes for all the three emotional conditions in depressed, compared to nondepressed, participants. According to previous literature, the frontal P2 reflects top-down matching between sensory inputs and stored memory traces (Luck and Hillyard, 1994; Federmeier et al., 2005; Evans and Federmeier, 2007; Freunberger et al., 2007). In this study, the larger P2 amplitude in depressed group may suggest excessive cognitive resources are allocated to the matching procedure in WM updating. Besides the P2, the LPP has been widely considered to reflect sustained attentional allocation and continuous processing of emotional stimuli (Johnston et al., 1986; Cuthbert et al., 2000; Weinberg and Hajcak, 2011; Hajcak et al., 2012; Auerbach et al., 2015; Lewis et al., 2015). In line with this notion, the LPP in this study may index the maintenance process during WM updating of emotional words. This idea is supported by Ruchkin et al. (1990) who found a correlation between the LPP and information maintenance in a WM task. The current finding that larger LPP in depressed patients indicates an excessive effort to maintain positive representation in this population. In line with our findings, previous studies using fMRI technique also revealed hyperactivity in frontal cortex and the anterior cingulate when depressed subjects performed n-back task with non-emotional stimuli (Harvey et al., 2005; Rose et al., 2006; Matsuo et al., 2007; Fitzgerald et al., 2008). It is proposed that a compensatory strategy (the usage of excessive cognitive resources and exertion of large mental effort) is needed for depressed individuals to perform relatively well in WM updating task, due to their low efficiency of executive control system. Although previous fMRI studies revealed an impairment in WM updating for depressed population, this study further revealed this deficit is associated with a low efficiency of matching and maintaining procedures in n-back task.

The current finding is not completely consistent with previously related studies. First, using the N-back paradigm, Levens and Gotlib (2010, 2015) found that depressed participants showed inferior performance both for positive and negative material. However, this study only found a poor response in positive condition. One possible reason for this inconsistency may be different methods of stimulus presentation. In the experiments of Levens and Gotlib (2010, 2015), participants were presented with faces of different emotion categories (positive, neutral, or negative) in a random order. By contrast, this study presented participants with a series of words with the same category in a block in one block. Second, Joormann et al. (2011) reported that in a WM manipulation task, depressed participants showed deficits when they manipulated negative but not positive words in WM. We suggest that the use of a different paradigm may be the reason of this inconsistency.

The limitation of the current study is that a 0-back task was not included so the absence of a baseline condition prevents us to exclude other cognitive deficits (e.g., visual perception, action planning and semantic comprehension) which may contribute to behavioral and ERP differences in the study.

In conclusion, the current study demonstrated a WM updating deficit specifically for positive material in depressed patients. This impairment is due to their insufficient selective attention to positive stimuli during memory encoding (reflected by reduced P1). Besides, when updating neutral and negative contents in WM, depressed patients consumed more cognitive resources in matching and maintaining processes (reflected by enhanced LPP and P2) to achieve similar task performance, as compared to nondepressed participants. Therefore, insufficient early attention to positive stimuli at memory encoding stage and low efficiency in matching and maintaining stages together lead to the deficits of WM updating in depression.

## Author Contributions

DZ and RG: conceived and designed the experiments. ZH and HX: performed the experiments. DZ: analyzed the data. DZ, HX and RG: wrote the manuscript. DZ and ZW: provided lab equipment for running the study.

## Conflict of Interest Statement

The authors declare that the research was conducted in the absence of any commercial or financial relationships that could be construed as a potential conflict of interest.
